# Prothrombin Gene Mutation as a Teaching Tool: An Autobiographical Case Report

**DOI:** 10.7759/cureus.33967

**Published:** 2023-01-19

**Authors:** Tucker Morris, Elizabeth R Lorbeer, Richard R Roach

**Affiliations:** 1 Department of Medical Education, Western Michigan University Homer Stryker M.D. School of Medicine, Kalamazoo, USA; 2 Department of Medical Library, Western Michigan University Homer Stryker M.D. School of Medicine, Kalamazoo, USA; 3 Department of Internal Medicine, Western Michigan University Homer Stryker M.D. School of Medicine, Kalamazoo, USA

**Keywords:** blood clot, cholecystectomy, pulmonary embolization, portal vein thrombosis, prothrombin g20210a factor ii mutation

## Abstract

The prothrombin G20210A factor II mutation carrier status has been reported to cause complications during pregnancy. This report presents the case of a patient diagnosed with heterozygous prothrombin G20210A factor II mutation at 29 years of age during preconception genetic screening. The patient had two uncomplicated pregnancies. The patient underwent laparoscopic cholecystectomy with a complicated postoperative course. The complications included deep vein thrombophlebitis (DVT), portal vein thrombosis (PVT), and pulmonary embolization (PE). The treatment options and contraceptive choices are also discussed in this report. Our report discusses the subsequent risks inherent in a heterozygote following the administration of oral contraceptives, prolonged immobilization related to lower extremity trauma, and extended motor vehicle excursion.

## Introduction

Prothrombin G20210A factor II mutation can cause life-threatening complications. The prevalence of heterozygous prothrombin G20210A factor II mutation is < 6% in Caucasians and < 1% in African and Asian populations [1]. The mutation has been reported to cause increased pregnancy loss and even maternal mortality [2]. Furthermore, the choice of using estrogen-containing contraceptives over progestin-only contraceptive methods in carriers of this mutation remains controversial in terms of safety. Progestin-only contraceptives have been shown to decrease the risk of venous thromboembolism relative to estrogen-containing contraceptives [2]. Prothrombin mutation alone is generally insufficient to cause venous thrombosis, particularly in heterozygotes. However, most patients are unaware of their prothrombin gene mutation status as most of them do not experience thrombotic events during their lifetime [3].

In addition to the effect on pregnancy and maternal health choices in patients with the mutation, there is a lifelong risk of thrombophlebitis, pulmonary emboli, and portal vein thrombosis triggered by immobilization, trauma, and surgery. This may be explained by the higher-than-normal prothrombin level seen in these patients, resulting in increased thrombotic risk. The prothrombin G20210A factor II mutation remains the second-most common cause of inherited thrombophilia in the United States [4].

This report presents the case of a patient with a heterozygous prothrombin G20210A factor II mutation, discusses the various complications experienced by the patient related to the mutation, and covers the different strategies used to manage and prevent further complications.

## Case presentation

**Figure 1:**
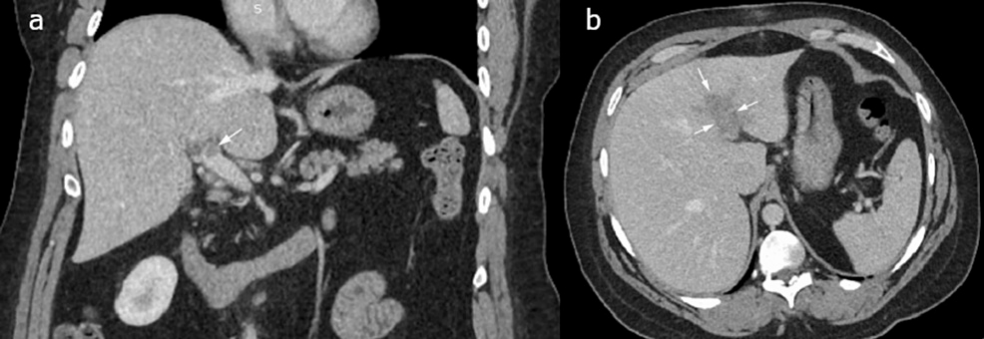
CT scan with contrast at portal venous phase revealed extensive thrombus extending from the main portal vein bifurcation (coronal CT, Figure 1A) into the left portal vein with periportal edema (axial CT, Figure 1B)

The patient (ERL) was diagnosed with a heterozygous prothrombin gene mutation at the age of 29 years through preconception genetic screening and laboratory tests. Following the diagnosis, oral contraceptive Desogen® (ethinyl estradiol and desogestrel) was discontinued as an estrogen-containing oral contraceptive, and Depo-Provera®, a medroxyprogesterone injection, was prescribed. This substitution was based on the increased risk of blood clots associated with estrogen-containing oral contraceptives [2]. After the birth of her two children (in 2008 and 2011), she was prescribed progestin-only contraceptives. At 41 years of age, the patient slipped and landed on her left knee. Three weeks later, she took an extended trip to visit family. Upon returning, her left leg “grew heavy." When she developed difficulty breathing, she sought emergency care. The emergency department diagnosed the patient with a deep vein thrombosis (DVT), and computed tomography (CT) pulmonary angiogram (Figure 1) revealed bilateral pulmonary emboli. The patient was administered an anticoagulant, warfarin, and oral contraceptives were discontinued. She continued warfarin therapy for nine months. Following the warfarin therapy, her primary physician switched her to aspirin (81 mg daily).

At 47 years of age, she underwent a laparoscopic cholecystectomy. Her surgeon administered low-molecular-weight heparin in the form of enoxaparin sodium (Lovenox) before surgery to manage potential thrombosis. No thromboprophylaxis was administered after surgery. However, 10 days post-surgery, the patient developed an occlusive left portal venous thrombosis and a non-occlusive thrombus in the right portal vein. The patient was treated with an intravenous bolus of 6,800 units of unfractionated heparin. She then received a heparin titration of 25,000 units at 1,400 units per hour until she was discharged 72 hours later. At discharge, she transitioned to long-term anticoagulation with 20 mg of rivaroxaban (Xarelto) for six months.

The patient remained at this dose for six months. After consulting with her hematologist, they agreed to decrease the dose to 10 mg since no other clotting events had occurred, and the patient desired to take the lowest efficacious dose possible.

## Discussion

We describe the case of a 47-year-old woman (ERL) who had two uncomplicated pregnancies despite being diagnosed with heterozygous prothrombin G20210A factor II mutation during preconception screening. During the next 18 years, she developed many previously reported complications associated with the mutation [2]. These complications included portal vein thrombosis following laparoscopic cholecystectomy. However, as of 2022, only six cases of portal vein thrombosis following cholecystectomy have been reported [5-10] (Table 1). Moreover, only one case has been reported on portal vein thrombosis following cholecystectomy associated with the prothrombin G20210A mutation factor II [8] (Table 1).

**Table 1:** Cases of Portal Vein Thrombosis After Cholecystectomy AST: aspartate aminotransferase; ALT: alanine aminotransferase; ALP: alkaline phosphatase; LFT: liver function tests; Hb: hemoglobin; PT: prothrombin time; eGFR: estimated glomerular filtration rate

Reference	Year	Age/Gender	Procedure	Medical Conditions	Post-operative Day	Physical Exam	Lab Tests	Imaging	Treatment
Brink et al. [5]	2003	12/F	Laparoscopic splenectomy and cholecystectomy	Autoimmune hemolytic anemia, Exercise-induced asthma	Sixteen	Diffuse right upper quadrant and epigastric tenderness without peritoneal irritation	Hb – 8.5	Doppler ultrasound (US) showed occlusive thrombus in the main, right, and left hepatic portal veins	Intravenous (IV) heparin for acute anticoagulation, switched to warfarin and anagrelide as an outpatient
							Lipase – normal		
							Amylase – normal		
							AST – normal		
							ALT – normal		
Rossi et al. [6]	2007	8/M	Laparoscopic splenectomy and cholecystectomy	Congenital hemolytic anemia	Six	Epigastric tenderness	Not specified	US, Doppler, and CT showed occlusive thrombus in the right main branch of the hepatic portal vein	Subcutaneous heparin and oral aspirin, oral anticoagulants on postoperative day 14
Dan et al. [7]	2011	63/F	Laparoscopic cholecystectomy	Hypertension, diabetes mellitus, ischemic heart disease, cholelithiasis	Four	Afebrile, anicteric, normal body habitus, non-tender abdomen	LFT – elevated	US showing ascites, CT scan showed PVT	IV fluids, antibiotics initially until Dengue fever infection cleared, started on enoxaparin on postoperative day ten
							WBC – 16,000		
							Dengue fever titers – positive		
Gul et al. [8]	2012	55/F	Laparoscopic cholecystectomy	Undiagnosed prothrombin G20210A factor II mutation	Sixty-one	Diffuse abdominal tenderness	WBC – 15,400	Abdomen X-ray revealed air fluid levels within the small bowel; abdominal CT and magnetic resonance angiogram (MRA) displayed portal and mesenteric vein thrombosis	Low molecular weight heparin (LMWH) followed by oral warfarin
							Amylase – normal		
							Lipase – normal		
							PT – 10.6 s		
							LFT - normal		
Ikoma et al. [9]	2014	31/F	Laparoscopic cholecystectomy	Cholelithiasis, hypertension	Ten	Minimal abdominal tenderness	AST – 466 U/L	US showed left portal vein PVT confirmed by abdomen CT	Started on enoxaparin, discharged with therapeutic LMWH and oral warfarin
							ALT – 271 U/L		
							ALP – 219 U/L		
							Total bilirubin – 1.1 mg/dL		
Sood et al. [10]	2022	43/F	Laparoscopic cholecystectomy	None	Twelve	Rigid and guarded abdomen with diffuse tenderness	ALP - 133 U/L	US exhibited some dilated small bowel loops indicative of PVT; contrast CT confirmed PVT at portal vein bifurcation	Subcutaneous enoxaparin, replaced by dabigatran for long-term anticoagulation
							D-dimer - 2309 ng/L		
							WBC - elevated		
							Thrombophilia - negative		
This case	2021	47/F	Laparoscopic cholecystectomy	Heterozygous prothrombin G20210A factor II mutation	Ten	Abdominal distension with tenderness	AST - 19 U/L	Abdomen and pelvis CT showed extensive occlusive thrombus in the left portal vein and a nonocclusive thrombus in the right portal vein	Heparin for acute anticoagulation, transitioned to rivaroxaban for long-term anticoagulation
							ALT - 19 U/L		
							ALP - 84 U/L		
							Lipase - 23 U/L		
							eGFR - 60 mL/min		

Prothrombin gene mutation alone is generally insufficient to cause venous thrombosis, particularly in heterozygotes [11]. However, potentially deadly blood clots may form in association with other contributing risk factors. Clots resulting from an identifiable inciting event including surgery and long periods of immobilization, such as hospitalization and travel, are considered to provoke clots [12]. The unprovoked clots reported in the literature are caused by unknown etiologies [12].

Blood clots, both provoked or unprovoked, influence a healthcare provider’s decision to initiate anticoagulation therapy and determine the duration of therapy. Current treatment guidelines recommend administering a direct oral anticoagulant (DOAC), such as apixaban, dabigatran, edoxaban, and rivaroxaban, in all patients with acute venous thromboembolism (VTE) [13]. The duration of this therapy should be at least three to six months. Lifelong treatment is advised for many patients with unprovoked VTE, even those without an inherited thrombophilia diagnosis [13].

The prothrombin G20210A factor II mutation poses a modest risk of VTE recurrence, even in heterozygotes. The presence of this mutation or carrier state alone may not warrant the side effects of indefinite antithrombotic therapy [14]; however, as our patient demonstrates, the risk is lifelong and potentially life-threatening. In such cases, prolonged treatment is advised after multiple thrombosis episodes.

Antithrombotic prophylaxis for patients with inherited thrombophilia undergoing surgery is more complicated. The American College of Chest Physicians published guidelines for perioperative management of antithrombotic therapy; however, post-operative guidelines are lacking. Patients with a history of VTE are at a significantly increased risk of recurrent VTE after surgery [15]. A study of patients who underwent laparoscopic colorectal surgery revealed that extending anticoagulation into the post-operative period decreases the rate of VTE with no increased risk of major bleeding [16].

## Conclusions

This case summarizes the course of a patient who developed a portal vein thrombosis after laparoscopic cholecystectomy with a diagnosed heterozygous prothrombin G20210A factor II mutation. Patients with inherited thrombophilia are at an increased risk of developing acute VTE after surgery and the literature supports continuing anticoagulation post-operatively to reduce VTE risk. This warrants further investigation into the benefits of antithrombotic prophylaxis following surgery for patients with inherited thrombophilia and reevaluation of the current approach.

The learner (TM) interest in internal medicine and hematology of this case would help physicians gain a perspective on detecting abnormalities in coagulation.

## References

[1] Zivelin A, Rosenberg N, Faier S (1998). A single genetic origin for the common prothrombotic G20210A polymorphism in the prothrombin gene. Blood.

[2] Varga EA, Moll S (2004). Cardiology patient pages. Prothrombin 20210 mutation (factor II mutation). Circulation.

[3] Bosler D, Mattson J, Crisan D (2006). Phenotypic heterogeneity in patients with homozygous prothrombin 20210AA genotype: A paper from the 2005 William Beaumont Hospital Symposium on Molecular Pathology. J Mol Diagn.

[4] Dautaj A, Krasi G, Bushati V (2019). Hereditary thrombophilia. Acta Biomed.

[5] Brink JS, Brown AK, Palmer BA, Moir C, Rodeberg DR (2003). Portal vein thrombosis after laparoscopy-assisted splenectomy and cholecystectomy. J Pediatr Surg.

[6] Rossi E, Michelini ME, Pignatti CB, Zanotti F, Franchella A (2007). A case of portal vein thrombosis after laparoscopy-assisted splenectomy and cholecystectomy in a child. J Pediatr Surg.

[7] Dan D, King K, Seetahal S, Naraynsingh V, Hariharan S (2011). Portal vein thrombosis following laparoscopic cholecystectomy complicated by dengue viral infection: a case report. J Med Case Rep.

[8] Gul W, Abbass K, Qazi AM, Markert RJ, Barde CJ (2012). Thrombosis of portal venous system after laparoscopic cholecystectomy in a patient with prothrombin gene mutation. JSLS.

[9] Ikoma N, Anderson CL, Ohanian M, Juneja HS, MacFadyen BV, Shah SK, Bajwa KS (2014). Portal vein thrombosis after laparoscopic cholecystectomy. JSLS.

[10] Sood R, Seth M, Kundal S, Singh R, Kapoor B (2022). A rare case of portal vein thrombosis following a successful laparoscopic cholecystectomy. Cureus.

[11] Kujovich JL (2021). Prothrombin thrombophilia. GeneReviews.

[12] Kearon C, Ageno W, Cannegieter SC, Cosmi B, Geersing GJ, Kyrle PA (2016). Categorization of patients as having provoked or unprovoked venous thromboembolism: guidance from the SSC of ISTH. J Thromb Haemost.

[13] Kearon C, Akl EA, Ornelas J (2016). Antithrombotic therapy for VTE disease: CHEST guideline and expert panel report. Chest.

[14] Ho WK, Hankey GJ, Quinlan DJ, Eikelboom JW (2006). Risk of recurrent venous thromboembolism in patients with common thrombophilia: a systematic review. Arch Intern Med.

[15] Nemeth B, Lijfering WM, Nelissen RG, Schipper IB, Rosendaal FR, le Cessie S, Cannegieter SC (2019). Risk and risk factors associated with recurrent venous thromboembolism following surgery in patients with history of venous thromboembolism. JAMA Netw Open.

[16] Vedovati MC, Becattini C, Rondelli F (2014). A randomized study on 1-week versus 4-week prophylaxis for venous thromboembolism after laparoscopic surgery for colorectal cancer. Ann Surg.

